# Adaptive Obstacle Detection for Mobile Robots in Urban Environments Using Downward-Looking 2D LiDAR

**DOI:** 10.3390/s18061749

**Published:** 2018-05-29

**Authors:** Cong Pang, Xunyu Zhong, Huosheng Hu, Jun Tian, Xiafu Peng, Jianping Zeng

**Affiliations:** 1 Department of Automation, Xiamen University, Xiamen 361102, China; 23220161151527@stu.xmu.edu.cn (C.P.); tianjun256@163.com (J.T.); xfpeng@xmu.edu.cn (X.P.); jpzeng@xmu.edu.cn (J.Z.); 2 School of Computer Science and Electronic Engineering, University of Essex, Wivenhoe Park, Colchester, Essex CO4 3SQ, UK; hhu@essex.ac.uk

**Keywords:** obstacle detection, outdoor mobile robot, LiDAR sensor, line segments, road height and vector

## Abstract

Environment perception is important for collision-free motion planning of outdoor mobile robots. This paper presents an adaptive obstacle detection method for outdoor mobile robots using a single downward-looking LiDAR sensor. The method begins by extracting line segments from the raw sensor data, and then estimates the height and the vector of the scanned road surface at each moment. Subsequently, the segments are divided into either road ground or obstacles based on the average height of each line segment and the deviation between the line segment and the road vector estimated from the previous measurements. A series of experiments have been conducted in several scenarios, including normal scenes and complex scenes. The experimental results show that the proposed approach can accurately detect obstacles on roads and could effectively deal with the different heights of obstacles in urban road environments.

## 1. Introduction

### 1.1. Motivation

With the rapid development of autonomous mobile robots used in outdoor environments such as delivery robots, self-driving street transporters and other unmanned ground vehicles (UGVs), the detection of traversable road regions and obstacles becomes an essential issue to guarantee their safe navigation [1,2]. It is critical for autonomous mobile robots to accurately perceive and understand the obstacles in front of them on the road surfaces when they are moving. Cameras were widely used for detecting roads, lane marks, obstacles and objects [3,4]. The visual sensors are very effective in scene understanding, but they are easily affected by light changes, and when it comes to complex shadows or bad weather conditions, the detection accuracy will be greatly reduced [5]. Therefore, cameras usually need to be combined with laser scanners to achieve high-accuracy information [6]. LiDAR sensors are widely used to detect the objects and obstacles due to their good range resolution and high accuracy [7]. For small UGVs used in urban areas, two important aspects should be considered: one is that the method of obstacle detection could be applicable to complex road environments, another is that these small UGVs must not require costly sensors as an economical solution is very important for their industrialization.

### 1.2. Related Works

In general, LiDAR sensors can be divided into two versions, namely 2D LiDAR and 3D LiDAR. 3D versions can obtain much richer information about the environment surrounding a mobile robot. Many object detection and classification applications have been done using 3D LiDAR [8,9,10,11]. Zermas et al. developed a 3D-LiDAR-based perception system for ground robots [12], which extracted the ground surface in an iterative fashion using deterministically assigned seed points so that the remaining non-ground points can be effectively clustered. In [13], they presented an algorithm for segmentation of 3D point clouds by establishing a binary labeling of scanned points and estimated the local ground plane to separate non-ground points, whereby the local ground plane was estimated by applying 2D line extraction algorithms to the domain of unorganized 3D points. However, the data obtained by 3D LiDAR is large and complicated, which results in more processing time. Moreover, the cost of 3D LiDAR is too expensive to be acceptable for small unmanned vehicles, therefore, 2D LiDAR sensors have been widely used for obstacle detection and terrain classification, because of their low cost.

Usually, a horizontally-looking 2D LiDAR mounted at a certain height on a mobile robot is used to detect obstacles or to judge traversable/untraversable areas in its near front. A method was proposed for dynamically detecting obstacles by using a horizontally-looking LiDAR based on an occupancy grid map [14], where the historic information of the map is used to decide whether a cell is occupied by a dynamic object. Chung et al. proposed an algorithm for detecting human legs and tracking them with a single laser range finder. The human legs were detected by the application of a support vector data description scheme and it tracked the leg positions according to an analysis of human walking motion [15]. Arras, et al. also used 2D laser range data and applied the AdaBoost algorithm for training a strong classifier to facilitate the detection of people [16]. However, these methods used only level 2D information, a restriction that limits their use for security applications. Because only a sliced sample of the world can be obtained in each scan, the obstacles that are lower than the scanning height cannot be detected by horizontally-looking 2D LiDAR sensors.

A downward-looking 2D LiDAR sensor can obtain more information about the road surface and detect frontal obstacles better than a horizontally-looking LiDAR sensor. It can recognize traversable road regions, obstacles with different height and other impassable areas such as holes and ditches. The famous Stanley autonomous driving car, which won the DARPA Grand Challenge, collected a scanned 3-D point cloud using downward-looking LiDAR sensors [17]. Qin, et al., used two 2D LiDAR sensors with different tilt-down angles to scan the frontal ground [18], but their methods for obstacles detection were not given. Lee and Oh proposed an approach for traversable region extraction in indoor/outdoor environments [19]. A quantized digital elevation map was firstly created using a grayscale reconstruction, and then the traversable region extraction was implemented with the histogram and edge information of this map. They adopted different classification methods to analyze the geographical features of different scenes, but did not discuss the conversion between different schemes under the environment changes. 

Usually, terrain classification and obstacle extraction procedures are based on the heights of the area contained in a digital elevation model, or use other derived models such as slopes and gradients [20,21]. Andersen, et al. proposed an algorithm for terrain classification that fused four distinctly different classifiers, i.e., raw height, step size, slope and roughness [22]. However, the terrain classifiers were all done on raw point statistics and the noise points were not considered. Zhang combined prior knowledge of the minimal width of roads and local-extreme-signal detection filter for separating the road segments and road-edge points, then the road-edges points were projected to the ground plane for further estimating the curb position [23]. Han, et al. presented a method to classify the extracted line segments into road and obstacles and the estimation of the roll and pitch angles of the sensor relative to the scanning road surfaces were used in this process [24]. In their approach, the change of roll and pitch of line segments were firstly used for judging obstacles and the criterion of road width was applied for selecting line segments corresponding to traversable or un-traversable roads. Finally, the obstacle height was used for making sure that the obstacle line segments were extracted exactly. 

Wijesoma et al. used a tilted 2D laser range finder to detect road curbs [25]. In this approach, the extracted curbs were tracked with a Kalman filter using successive scans and the prior knowledge assumptions was used for finding the right curbs. Liu et al. created a local digital elevation map with the average height of points in voxels. Candidate road curbs were extracted based on the height variance and slope of two adjacent voxels [26]. Although these algorithms use downward-looking LiDAR sensors and have achieved some good experimental results, they do not deal with complex curved slopes and are poorly suited for changing road conditions, such as sloping roads with sharp turns. Table 1 provides an overview of the major terrain classification and obstacle extraction algorithms described in the aforementioned works.

In many cases, the extracted features are more useful and effective than raw data. The detected straight lines and corners are used to match with the global straight lines and corners to get the robot position and orientation values [27]. Three geometric primitives, lines, circles and ellipses, were discussed for 2D segmentation [28]. Zhao, et al. presented a prediction-based geometrical feature extraction approach which worked independent of any prior knowledge of the environment to detect line and circle features from 2D laser scanner data [29]. Among these geometric primitives, line extraction is the simplest one owe to its simplicity and easy implementation. A method based on scan lines was reported to extract road marking [30], in which the road points were separated from the raw point clouds using the seed road points by moving least squares line fitting. The method of total least squares to fit a line was discussed in [31]. Sarkar et al. proposed an offline method to build maps of indoor environments by using line segments extracted from laser range data [32]. Several algorithms had been proposed for extracting line segments from 2D LiDAR data, Improved Successive Edge Following algorithm [33], Recursive Line Extraction algorithm [34] and qualitative and quantitative comparisons had been applied using different methods include Line Tracking, Iterative End-Point Fit (IEPF) and Split and Merge Fuzzy algorithms [35], IEPF is used in this paper, because it is simple and efficient. In these algorithms, obstacles were referred to objects above the road surface. The heights of the detected objects were determined for obstacles avoidance. 

### 1.3. The proposed Approach

In order to improve the applicability for complex road environments, this paper proposes a new obstacles detection method by using a single downward-looking 2D LiDAR sensor. The method is mainly depends on the line segments extraction from the LiDAR data, as well as the adaptive estimation of the height and vector of the scanned road surface. Our approach is novel in terms of the following three aspects in comparison with other existing methods based on 2D LiDAR:

(1) Most previous studies did not deal with complex slopes and are poorly suited to different road conditions only using road height estimation. In order to improve the accuracy, we define the road vectors to well reflect the real situation of a road. We divide the line segments into ground and obstacle sections based on the average height of each line segment and the deviation of the line segment from the scanned road vector estimated from the previous measurements. By combining the height and the vector of the scanned road, our method can adapt to different road conditions.
(2) The estimated road height and road vector in our method can vary with the changing road conditions, which improves the adaptivity of our method. The most recent characteristics of the road can be learnt by estimating the height and the vector a set of past scanned laser data, and then used for separation of obstacles at the next moment. The entire process is conducted iteratively so that a self-supervised learning system is realized to cope with uphill road, downhill road and sloping road.
(3) We need not measure or estimate the roll and pitch angles of the LiDAR. During the whole design and application process, only the 2D planar position information and the steering angle of the robot are used, but we can also detect the road conditions and obstacles whether the robot is on an uphill road or a downhill road. The whole structure is simple and the algorithms are low time-consuming and applicable to small unmanned vehicles effectively in urban outdoor environments.

The rest of this paper is organized as follows: Section 2 presents the definitions of the system coordinates used in this work. The method for extracting line segments from the scanned points is illustrated in Section 3. Section 4 describes our new algorithms used for obstacle detection based on the height estimation and vector extraction of each scanned road surface. Several typical experiments are conducted and the results are analyzed in Section 5. Finally, a brief conclusion and future work suggestions are given in Section 6.

## 2. Definitions of System Coordinates

**Figure 1:**
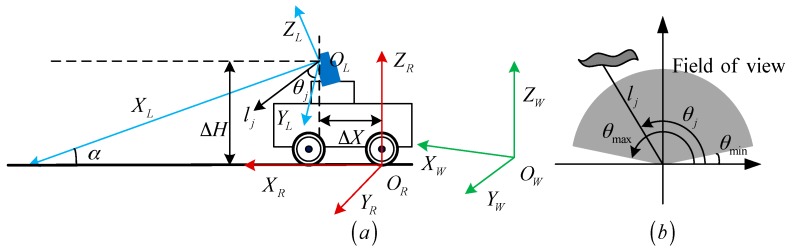
Definitions of the system coordinates. (**a**) Coordinate system; (**b**) Laser polar coordinate system.

As shown in Figure 1a, the laser scanner is tilted at angle *α* down towards the ground. We define the laser frame $F_{L}\left(O_{L},\theta ,l,X_{L},Y_{L}\right)$ ($O_{L}$ is the laser emission point). The scan starts at direction $\theta_{\min}=\theta_{1}$ and stops at $\theta_{\max}=\theta_{N}$ (Figure 1b) with a given angular resolution $\Delta \theta =\theta_{j}-\theta_{j-1}$. The robot frame is defined as $F_{R}\left(O_{R},X_{R},Y_{R},Z_{R}\right)$ ($O_{R}$ is the point of contact between the rear wheel and the ground). The world frame is defined as $F_{W}\left(O_{W},X_{W},Y_{W},Z_{W}\right)$ ($O_{W}$ is the rear wheel position point at the initial moment $t_{0}$). $F_{L},F_{R},F_{W}$ are shown with blue, red and green, respectively.

**Figure 2:**
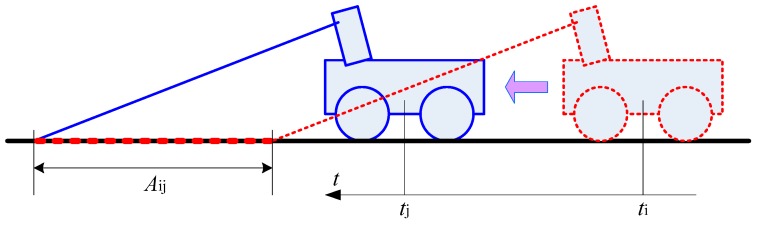
The scanned area in front of the robot.

The 2D pose vector of the mobile robot is $\left(x_{t_{i}},y_{t_{i}},\beta_{t_{i}}\right)$, where $\left(x_{t_{i}},y_{t_{i}}\right),\beta_{t_{i}}$ are the position and heading angle of mobile robot in the world coordinate system at time $t_{i}$. Many researchers use odometers or IMUs to get robot position information, which have some measurement errors. In our method, we have performed some filtering to reduce the errors which may be caused by tire deformation or tire slip. As shown in Figure 2, because we only detect obstacles in the area $A_{ij}$ ahead of the robot (scanned for time $t_{i}$ to $t_{j}$) and not pay attention to the scanned road where the robot has passed, the accumulation errors of odometer in the time span between $t_{i}$ and $t_{i}$ is small and can be ignored. We define the data of a 2D LiDAR scan at time $t_{i}$ as:

(1) $$P_{t_{i}}^{L}=\left\{\left(\theta_{ij},l_{ij},x_{ij}^{L},y_{ij}^{L}\right)\right\}\;1\leq j\leq N$$

(2) $$x_{ij}^{L}=l_{ij}\cos\theta_{ij},y_{ij}^{L}=l_{ij}\sin\theta_{ij}$$

where $\left(\theta_{ij},l_{ij}\right)$ are the polar coordinates of the current j-th point in $F_{L}$, $\theta_{ij}$ represents the angle and $l_{ij}$ is the distance of this point, and $\left(x_{ij}^{L},y_{ij}^{L}\right)$ is its Cartesian coordinates in $F_{L}$. i represents the current time $t_{i}$. The conversion from the laser Cartesian coordinate system to the global Cartesian coordinate system is written as follows:

(3) $$\left[\begin{array}{c} x_{ij}^{R}\\ y_{ij}^{R}\\ z_{ij}^{R} \end{array}\right]=R_{t_{i}}^{R}\cdot \left[\begin{array}{c} x_{ij}^{L}\\ y_{ij}^{L}\\ 0 \end{array}\right]+\left[\begin{array}{c} \Delta X\\ 0\\ \Delta H \end{array}\right]$$

(4) $$\left[\begin{array}{c} x_{ij}^{W}\\ y_{ij}^{W}\\ z_{ij}^{W} \end{array}\right]=R_{t_{i}}^{W}\cdot \left[\begin{array}{c} x_{ij}^{R}\\ y_{ij}^{R}\\ z_{ij}^{R} \end{array}\right]+\left[\begin{array}{c} x_{t_{i}}\\ y_{t_{i}}\\ 0 \end{array}\right]$$

where $\left(x_{ij}^{W},y_{ij}^{W},z_{ij}^{W}\right)$ and $\left(x_{ij}^{R},y_{ij}^{R},z_{ij}^{R}\right)$ respectively represent the coordinates in $F_{R}$ and the global coordinates of the current j-th point in $F_{W}$. $R_{t_{i}}^{R}$ is the rotation transformation matrix from $F_{L}$ to $F_{R}$, and $R_{t_{i}}^{W}$ is the rotation transformation matrix from $F_{R}$ to $F_{W}$.

## 3. Scan Segmentation and Line Extraction

Scan segmentation is the first step for a robot to detect its environment, which divides the scanned points, and then uses linear segments to fit them. Each line segment will be classified as obstacle segment or pavement segment. The range data received from 2D LiDAR at $t_{i}$ are in polar coordinate form. All points are in a plane and each point has its own index number (ij), and their coordinates $\left(\theta_{ij},l_{ij}\right)$ or $\left(x_{ij}^{L},y_{ij}^{L}\right)$ are two-dimensional in $F_{L}$. The processing in $F_{L}$ is much faster than in the 3D coordinates $\left(x_{ij}^{W},y_{ij}^{W},z_{ij}^{W}\right)$ in $F_{W}$. For this reason, the scan extraction has been done in laser coordinates. After segmentation, each line is converted to the $F_{W}$ using Equations (3) and (4) according to the indices of its first and last points.

### 3.1. Breakpoint Detection

A rupture point indicates the discontinuity or break in a series of points. Breakpoint detection is an important task for scan segmentation, and in a real environment, such a discontinuity always occurs where an obstacle appears. According to Equation (1), an initial segmentation for $P_{t_{i}}^{L}$ can be defined as:

(5) $$S_{iT}=\left\{\left(\theta_{ik},l_{ik}\right),n_{T}<k<n_{T+1}\right\},1<T<m$$

The point cloud is separated into *m* parts. The traditional method of segmentation is to separate the point cloud with a constant threshold $D_{th}$. However, it is difficult to determine the threshold, and the fixed threshold is less flexible. In the Adaptive Breakpoint Detector (ABD) [35], an adaptive threshold is adopted as:

(6) $$D_{th}=l_{ij}\cdot \frac{\sin\left(\Delta \theta \right)}{\sin\left(\lambda -\Delta \theta \right)}+3\sigma_{l}$$

where λ is an auxiliary parameter and $\sigma_{l}$ is a residual variance to encompass the stochastic behavior of the sequence scanned points $P_{t_{i}}^{L}$ and the related noise associated to $l_{ij}$. This threshold depends on the range scan distance $l_{ij}$, which is more flexible than a constant threshold and can be used in various situations for breakpoint detection.

The preliminary fragment and breakpoint detection is defined as follows:

(7) $$p_{i(j+1)}\in \left\{ \begin{array}{ll} S_{iT} & if\;\left\|p_{i(j+1)}-p_{ij}\right\|<D_{th}\\ S_{i(T+1)} & p_{ij},p_{i(j+1)}\to breakpoints\;else \end{array}\right.$$

where $p_{ij}$ is the j-th scanned point at $t_{i}$, $\left\|p_{i(j+1)}-p_{ij}\right\|$ is the Euclidean distance between two consecutive scanned points $p_{i(j+1)}$ and $p_{ij}$.

### 3.2. Line Extraction

**Figure 3:**
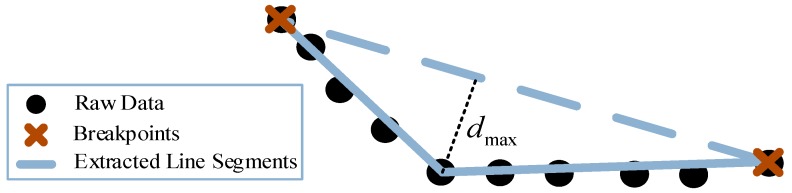
The IEPF algorithm principle.

Each point segment $S_{iT}$ with points less than κ is deleted to make sure that no error points are included in the segments. Then the IEPF algorithm [35] is used for separation of line extracted segments as shown in Figure 3. Finally, we get h lines at the global coordinate system by using Equation (8) below:

(8) $$L_{t_{i}}=\left\{l_{i1},\ldots ,l_{ih}\right\},\;l_{it}=\left\{\left(x_{k}^{W},y_{k}^{W},z_{k}^{W}\right),s_{it}<k<e_{it}\right\},1<t<h$$

where $s_{it},e_{it}$ are the order of the starting point and the end point of $l_{it}$.

After the segmentation algorithm, a complete sequence of the scanned points $\left(P_{t_{i}}^{L}\right)$ becomes a pair of $t^{th}$ values $\left\{\left(s_{it},e_{it}\right)\right\}$ which represent both end points of each line.

For each line, we extract its properties as follows:

(9) $$T=\left(h_{it},Sp_{it}^{W},Ep_{it}^{W},Sp_{it}^{L},Ep_{it}^{L},{\vec{v}}_{it},len_{it}\right)$$

$h_{it}$: The average height of $l_{it}$. $h_{it}=mean\left\{\left(z_{k}^{W}\right),\;s_{it}<k<e_{it}\right\}$
$Sp_{it}^{W}$: The starting point of $l_{it}$ in $F_{W}$. $Sp_{it}^{W}=\left(x_{s_{it}}^{W},y_{s_{it}}^{W},z_{s_{it}}^{W}\right)$
$Ep_{it}^{W}$: The end point of $l_{it}$ in $F_{W}$. $Ep_{it}^{W}=\left(x_{e_{it}}^{W},y_{e_{it}}^{W},z_{e_{it}}^{W}\right)$
$Sp_{it}^{L}$: The starting point of $l_{it}$ in $F_{L}$. $Sp_{it}^{L}=\left(x_{s_{it}}^{L},y_{s_{it}}^{L}\right)$
$Ep_{it}^{L}$: The end point of $l_{it}$ in $F_{L}$. $Ep_{it}^{L}=\left(x_{e_{it}}^{L},y_{e_{it}}^{L}\right)$
${\vec{v}}_{it}$: The vector of $l_{it}$. ${\vec{v}}_{it}=Ep_{it}^{W}-Sp_{it}^{W}$
$len_{it}$: The length of $l_{it}$. $len_{it}=\left\|Ep_{it}^{L}-Sp_{it}^{L}\right\|$

## 4. Obstacles Detection Algorithms

The main content of obstacle detection is to divide the line segments into either obstacles or road sections. For this purpose, the height $\left(height\left(t_{i}\right)\right)$ and the vector $\left({\vec{V}}_{t_{i}}\right)$ of each scanned road surface are estimated and extracted so that pavement information can be effectively obtained. Then, the obstacles and the road surfaces can be separated base on these two parameters.

**Figure 4:**
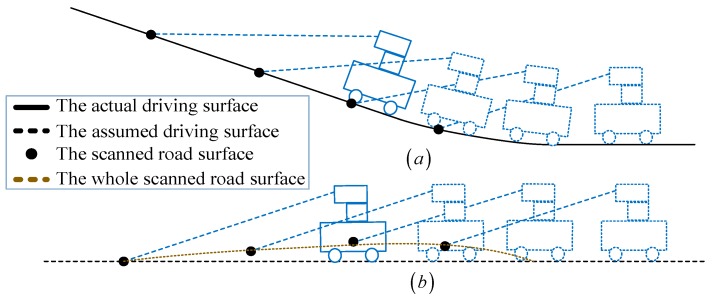
Obstacle detection process. (**a**) The actual moving surface; (**b**) The assumed moving surface.

Figure 4 shows the obstacle detection process, in which Figure 4a is the actual moving surface of the robot. Since no sensor is used to get the pitch and roll data of the robot relative to the world coordinate system, we only use a 2D pose vector $\left(x_{t_{i}},y_{t_{i}},\beta_{t_{i}}\right)$ of the mobile robot in the algorithms. The mobile robot is equivalent to moving on a hypothetical plane shown in Figure 4b. The dashed red line corresponds to the scanned road surface, although it is not the same as the actual moving surface. This still can reflect the real situation of the obstacles on pavement.

### 4.1. Road Height Estimation

When the mobile robot is moving forward, we estimate the height of the scanned road surface for each scan. This is extremely important for the mobile robot to identify obstacles on the pavement. We assume that the mobile robot usually begins moving on a flat road and no obstacles are in front of it. Therefore, the height $hight\left(t_{0}\right)$ of the scanned road surface corresponding to the first scan (at initial time $t_{0}$) can be derived from the average height of the laser points belong to central range (e.g., from 75° to 105°). This range corresponds exactly to the front of the robot. For $t_{i}>t_{0}$, the data from wider range (e.g., from 30° to 150°) are used to assess the $height\left(t_{i}\right)$, where most of the points in this scanned area are belong to ground. We need to filter out some obstacle points that may exist to get a more accurate height.

Since the height of the ground surface does not change suddenly, the height $\left(z_{ij}^{W}\right)$ of each point in the selected range is compared with the height of the scanned road estimated before. If the difference between a point’s height and the estimated height exceeds a threshold $\delta_{th}$, then this point will be removed. After filtering all obstacle points in this range, the height of this scan is calculated based on the remaining points. The estimated height varies with the different road conditions, it improves the adaptability of the whole algorithm. Algorithm 1 describes the algorithm proposed for assessing the height of the scanned road surface.

**Algorithm 1:** Height assessment of the scanned road surface.**INPUT**: all point $p_{ij}$ in range of (30°~150°) and $t_{i}$. **OUTPUT**: $height(t_{i})$ Ω = ∅ (*set* Ω *inital value is null set*) 01 *if*
*t_i_* = *t_0_* 02  *hight* (*t_i_*) = *average*
$\left(z_{ij}^{W}(from75{}^{\circ}to105{}^{\circ})\right)$ 03 *else* 04  *for all point p_ij_ in range of* (30°~150°) 05   $if\;abs(z_{ij}^{W}-hight\left(t_{i-1}\right))<\delta_{th}$ 06    $\Omega =\Omega \cup z_{ij}^{W}$ 07   *end* 08  *end* 09 *end* 10 $height(t_{i})=average(\Omega )$ **return**
*height* (*t_i_*)

### 4.2. Road Vector Extraction

In order to enhance the robustness and classification performance of the whole method, the vector ${\vec{V}}_{t_{i}}$ corresponding to the current scan is extracted by the line segments which are separated from the scanned points. It can be used for better fitting the road information. As the road is continuous, it will not change drastically in one scanning period. ${\vec{V}}_{t_{i-1}}$ obtained at the previous moment can be used to approximate the currently scanned road surface. By using this feature, the obstacles could be reliably detected.

Since the robot is originally moving on an unobstructed road, the main line segments corresponding to the first frame can be approximated to the scanned road surface. Let ${\vec{V}}_{t_{0}}$ be the longest line of the first scan:

(10) $${\vec{V}}_{t_{0}}={\vec{v}}_{t^{*}},t^{*}=\max\left(len_{it}\right)$$

When $t_{i}>t_{0}$, we use the line segment set $RL_{i}$ of the current scan, obtained in Section 4.3 (Algorithm 3), to get ${\vec{V}}_{t_{i}}$ after filtering the line segments.

Each extracted line segment in $RL_{i}$ has two attributes: direction angle and length. A line used to fit the vector of the scanned road surface should have low deviation of direction angle $\vartheta_{it}$ Equation (11) relative to ${\vec{V}}_{t_{i-1}}$:

(11) $$\vartheta_{it}=\arccos\left(\frac{{\vec{v}}_{it}\cdot {\vec{V}}_{t_{i-1}}}{\left\|{\vec{v}}_{it}\right\|\cdot \left\|{\vec{V}}_{t_{i-1}}\right\|}\right)$$

The line segment that is longer than a minimum line length $L_{\min}$ is more suitable for fitting the road vector. Moreover, a line segment is removed from $RL_{i}$ if its deviation of direction angle is bigger than its maximum threshold $\phi_{\max}$ or its length is shorter than $L_{\min}$. This criterion can effectively filter out some noisy lines caused by LiDAR and the robot localization, which leads to more accurate result and faster computation.

For the remaining line segments, their starting points $Sp_{it}^{L}$ and end points $Ep_{it}^{L}$ are extracted to Θ. The points are finally fitted into a straight line using the least square method. For this line, we choose two endpoints $p_{1}^{L},p_{2}^{L}$ and convert them to $F_{W}$, so that we can get ${\vec{V}}_{t_{i}}$. The estimated vector can well describe different road conditions including the sloping road and enhances the robustness of our method. The algorithm for extracting the vector of scanned road surface is described in Algorithm 2.

**Algorithm 2:** Extract the vector of scanned road surface.**INPUT**: road line segments sets $RL_{i}$, $\vartheta_{it}$ and $t_{i}$. **OUTPUT**: ${\vec{V}}_{t_{i}}$ Θ=∅ (set Θ inital value is null set) 01 $if\;t_{i}=t_{0}$ 02  $t^{*}=\max(len_{it})$ 03  ${\vec{V}}_{t_{i}}={\vec{v}}_{t^{*}}$ 04 *else* 05  $for\;all\;l_{it}\;in\;RL_{i}$ 06   $if\;\vartheta_{it}<\phi_{\max}\;and\;len_{it}>L_{\min}$ 07   $\Theta =\Theta \cup \left\{Sp_{it}^{L},Ep_{it}^{L}\right\}$ 08   *end* 09  *end* 10  $Get\;\hat{a},\hat{b}\;using\;the\;least\;square\;from\;\Theta$ 11  $choose\;two\;endpoints\;p_{1}^{L},\;p_{2}^{L}\;from\;line:y=\hat{a}x+\hat{b}$ 12  $Convert\;p_{1}^{L},\;p_{2}^{L}\;to\;F_{W}\;combining\left(3\right),and\;get\;p_{1}^{W},\;p_{2}^{W}$ 13  ${\vec{V}}_{t_{i}}=p_{2}^{W}-p_{1}^{W}$ 14 *end* **return**
${\vec{V}}_{t_{i}}$

### 4.3. Obstacle Extraction

Obstacle extraction is mainly based on two features: the average height of each line segment and the deviation of the line segment from the vector of the scanned road surface extracted. In many cases, obstacles are the objects and road edges that are higher than ground—thus the obstacle segments are usually the lines which deviate from the road surface.

Before obstacle extraction, some noisy segments need to be filtered and eliminated, which are grouped together by a number of points, like dirty spots. Only a threshold $l_{\min}$ can be used to filter them out. For each obstacle line, its $h_{it}$ should be bigger than a minimum threshold $\xi_{h}$, but the line segments whose $h_{it}$ are larger than $\xi_{h}$ may not be obstacle line segments. 

For example, when the mobile robot is running on a flat road, its laser has been scanned to the ramp of uphill road. So, we need to consider using the deviation between $l_{it}$ and ${\vec{V}}_{t_{i-1}}$ to make judgments. Here, the distances between the endpoints of line segment $l_{it}$ and the road vector ${\vec{V}}_{t_{i-1}}$, i.e., $D\left(Sp_{it}^{W},{\vec{V}}_{t_{i-1}}\right)$ and $D\left(Ep_{it}^{W},{\vec{V}}_{t_{i-1}}\right)$, are calculated. As long as one of these two distances is greater than a minimal deviation value $\xi_{i}$, the line segment will be treated as an obstacle line segment. The detailed algorithm for obstacles extraction is described as Algorithm 3.

In Algorithm 3, $OL_{i},RL_{i}$ denote the obstacle and the road line segment sets at current time $t_{i}$ respectively. For $\xi_{i}$, it is expressed by the following formula:

(12) $$\xi_{i}=\Delta t\times V_{i}+3\varsigma$$

where $\Delta t=t_{i+1}-t_{i}$ is the scanning interval of the LiDAR sensor, $V_{i}$ is the current speed of the robot and ς is deviation.

**Algorithm 3:** Obstacles extraction.**INPUT**:  the average height of line $h_{ij}$, ${\vec{V}}_{t_{i-1}}$ and $t_{i}$. **OUTPUT**: $OL_{i}\;and\;RL_{i}$ $OL_{i}=RL_{i}=\emptyset$ 01 $when\;t_{i}>t_{0}$ 02  $if\;len_{it}>l_{\min}$ 03   $if\;\left|h_{ij}\right|>\xi_{h}$ 04   $if\;D\left(Sp_{it}^{W},{\vec{V}}_{t_{i-1}}\right)>\xi_{i}\;or\;D\left(Ep_{it}^{W},{\vec{V}}_{t_{i-1}}\right)>\xi_{i}$ 05    $OL_{i}=OL_{i}\cup l_{ij}$ 06   *end* 07   $RL_{i}=RL_{i}\cup l_{ij}$ 08  *end* 09  $RL_{i}=RL_{i}\cup l_{ij}$ 10  *end* 11 *end* **return**
$OL_{i}\;and\;RL_{i}$

## 5. Experimental Results

**Figure 5:**
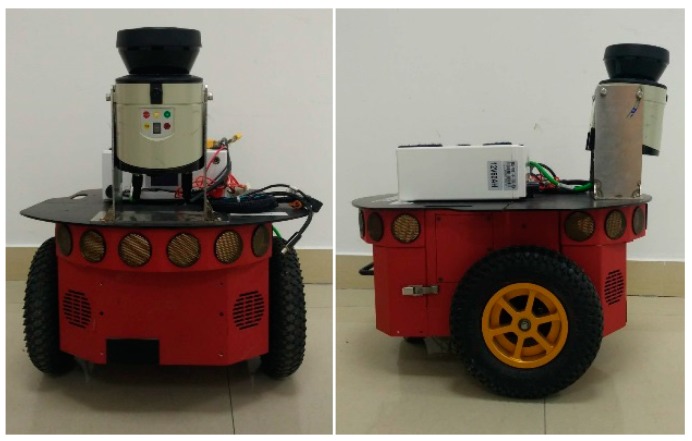
‘Pioneer3’ mobile robot.

To verify the effectiveness of our obstacles detection method, a series of experiments are implemented in urban outdoor environments using a ‘Pioneer3’ mobile robot (Figure 5), which is equipped with a Sick LMS111 LiDAR; The algorithms are tested using Matlab running on an Intel(R) Core(TM) i5-3470 computer. During the experiments, only the 2D planar position information and the steering angle of the robot are used, which are recorded from the odometer localization of the ‘Pioneer3’. The configuration parameters of the LiDAR sensor in the obstacles detection process are given in Table 2.

### 5.1. Parameters Analysis and Turning

In the proposed obstacles detection method, there are some parameters should be determined. The value of parameters λ and $\sigma_{l}$ are referenced from [35]. The parameter κ represents the minimum number of point in each segment $S_{iT}$ and can be used for removing error points from the segments. A small value should be set for $l_{\min}$ to eliminate the noisy segments. The height threshold of point $\delta_{th}$ is used to look for scanned points which belong to the road surface, and the height of the scanned road can be estimated from these points. This value is determined by the minimal height of obstacle we want to detect. In the process of vector extraction, the threshold $\phi_{\max}$ and $L_{\min}$ are used to find for line segments which belong to road surface. A bigger $\phi_{\max}$ and a small $L_{\min}$ may result in more points in Θ, but the accuracy of the estimated vector may be reduced. A small $\phi_{\max}$ and a bigger $L_{\min}$ may lead to no points in the current Θ and make the current vector extraction fail. The threshold $\xi_{h}$ is used for obstacles extraction, its value should match $\delta_{th}$. And the value of ς must be smaller than the chassis height and travel capacity of the mobile robot.

After a large number of tests, the parameters of the algorithms were carefully turned, their value shown in Table 3. In the practical application, these parameters need not to be changed once they were determined by test experiments.

### 5.2. Application Tests in Most Common Road Environment

**Figure 6:**
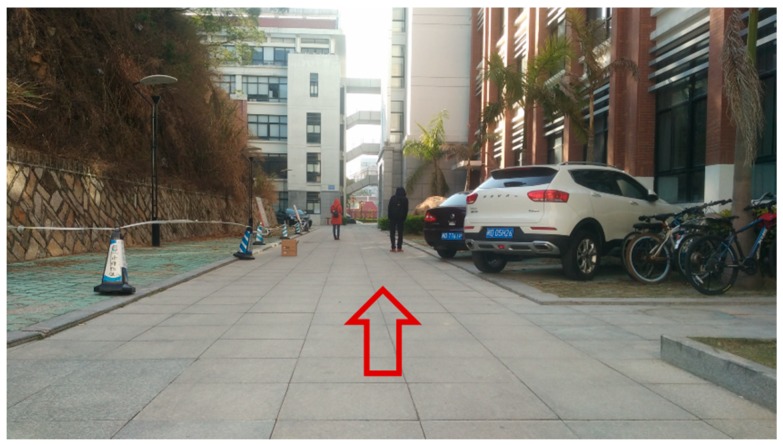
Scene 1—normal road.

**Figure 7:**
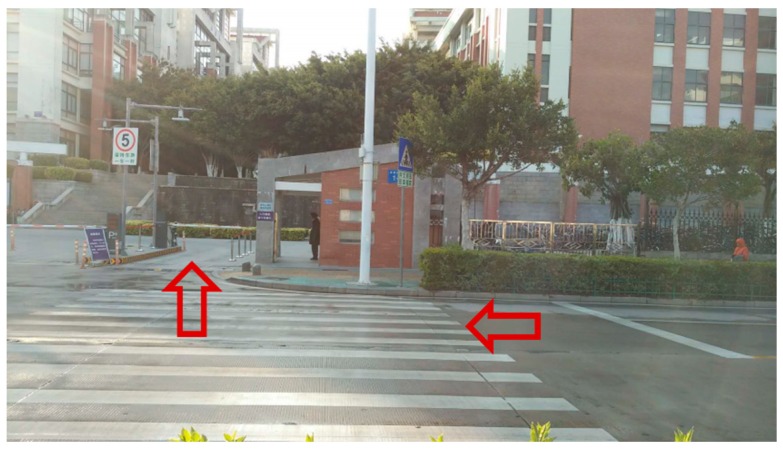
Scene 2—school gate.

Our testing was conducted within the two outdoor environments shown in Figure 6 and Figure 7. As can be seen, Figure 6 shows the scene of a normal road where the mobile robot was moving forward. At the right of the road, there is a flower bed, two cars parked on side, as well as few bicycles. In the middle of the road, two pedestrians and a box stood there, some road blocks on the left. On both sides of the road, there are other tall obstacles, such as buildings. Figure 7 shows the scene of a school gate, where the robot was moving along the right side of the road (nearby the flower bed) and turned right to enter the school gate. From this picture, we can see that the right shoulder of the road is impassable, and there are obstacles with different height on both side of the campus gate.

For Scene 1 (Figure 6), 1140 frames of LiDAR scanning data are obtained with the movement of the robot, and the laser-point cloud data are displayed in X-Y plane. 

**Figure 8:**
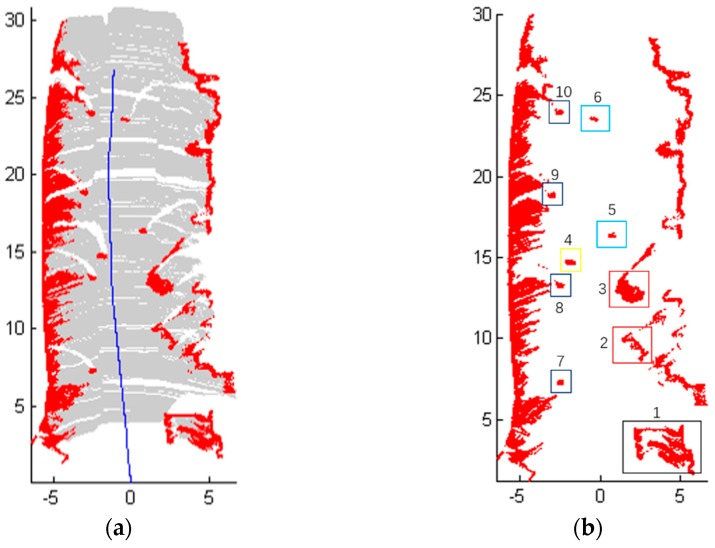
The detection results of Scene 1. (**a**) Results of obstacles detection; (**b**) The position of some objects which are detected.

Figure 8 shows the detection results of Scene 1. More specifically, Figure 8a shows the results of obstacles detection, where the blue line represents the track of the mobile robot, which is recorded from the odometer localization of the robot. Red points represent the obstacles and gray areas are the travelable area. Furthermore, it can be seen clearly in Figure 8b that the flower bed on the right is marked as ‘1’ and the cars on the right have been detected and marked as ‘2’ and ‘3’. The box is marked as ‘4’ and the two people in the middle of the road are detected as ‘5’ and ‘6’. Moreover, ‘7’‘8’‘9’‘10’ are the road blocks on the left. The road surface and obstacles on both sides have been detected too.

**Figure 9:**
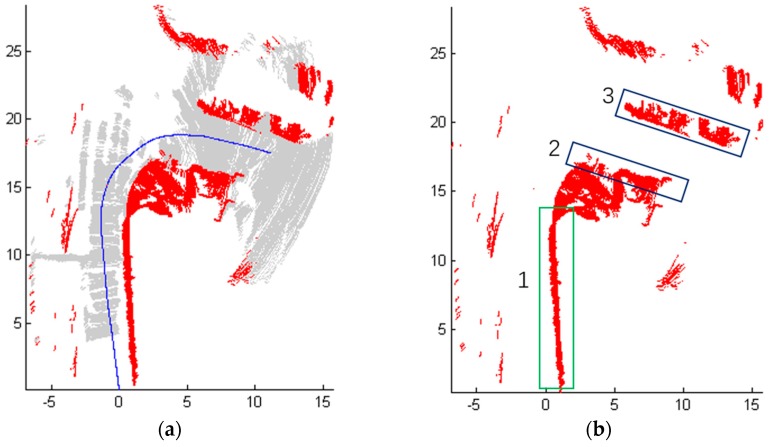
Obstacle detection results of Scene 2. (**a**) Results of obstacles detection; (**b**) The position of some obstacle area.

For the Scene 2 (Figure 7), 1350 frames of LiDAR scanning data were obtained when the mobile robot was moving, and the laser-point cloud data are displayed in X-Y plane. The detection results of Scene 2 are shown in Figure 9. Note that Figure 9a is the results of obstacles detection, in which the blue line represents the track of the mobile robot, red points represent the obstacles and the gray area is the travelable area. As shown in Figure 9b, the green rectangle area ‘1’ marks the detected right shoulder of the road, and Areas ‘2’ and ‘3’ are the obstacles on both sides of the gate.

### 5.3. Application Tests in Rare Complex Road Environment

**Figure 10:**
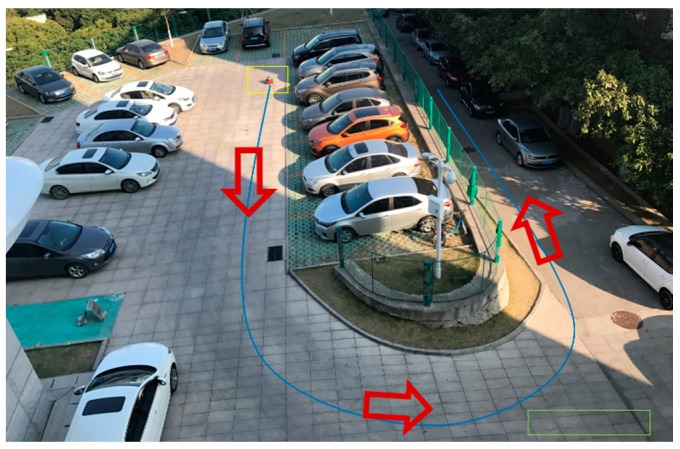
Scene3—a complex road with curve, downhill and slope.

**Figure 11:**
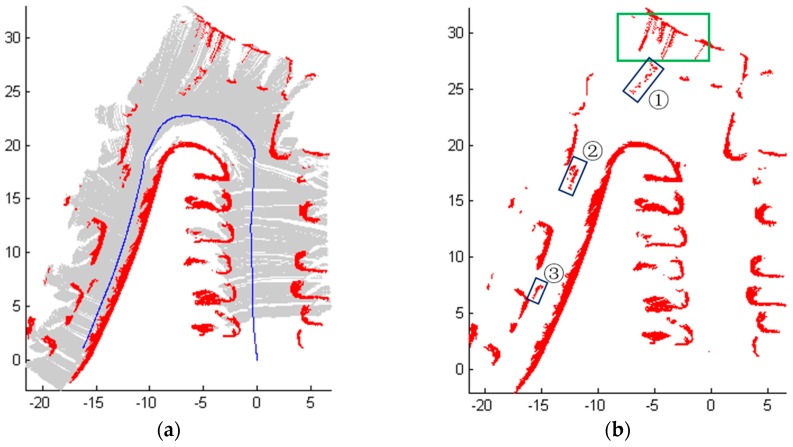
The detection results of Scene 3 with the joint using of the estimated height and the estimated vector. (**a**) Results of obstacles detection; (**b**) The position of some passerby obstacle areas.

Scene 1 and Scene 2 are relatively simple. In order to test our method, the mobile robot operates in a complex environment. As shown in Figure 10, the path contains a curve and a downhill, and the road surface is sloped. The mobile robot started from the position at the yellow rectangle and followed the blue path to move forward. For Scene 3 (Figure 10), 2500 frames of LiDAR scanning data were obtained when the mobile robot was moving. Figure 11 shows the detection results of Scene 3. 

More specifically, Figure 11a shows the outlines of the cars and all vehicles have been detected, and Figure 11b shows some obstacle blocks identified as ①–③ being detected, which were the pedestrians passed in front of the mobile robot. A green rectangle in Figure 10 was falsely detected as obstacles as this area is inclined and cannot be detected. Nevertheless, the un-detected obstacle area on the right side was far from the track of the robot and had no disturbance to the movement of the robot.

**Figure 12:**
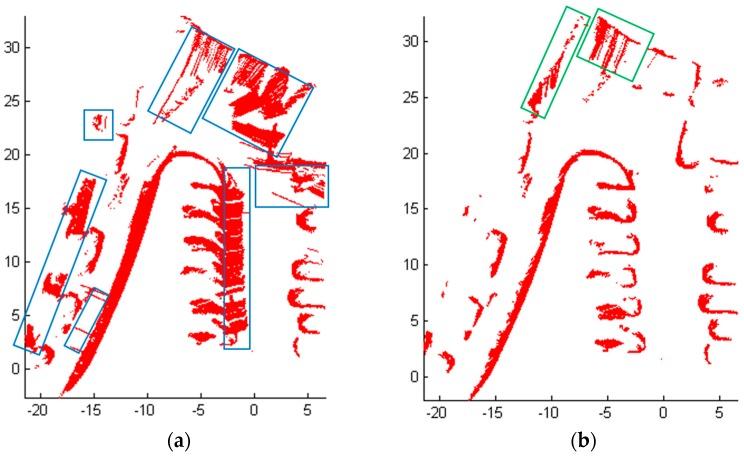
The comparison of detection results of Scene 3. (**a**) Using only the estimated vector; (**b**) Using only the estimated height.

The estimated height varies with the different road conditions and the estimated vector can well describe different road conditions including some rare complex road environments such as scene 3, they improve the adaptability and robustness of the whole algorithm. However, the detection results of Scene 3 using only the estimated height or the estimated vector are shown in Figure 12. A large sum of road area in blue boxes are detected mistakenly as obstacles in Figure 12a using only the estimated vector. And as shown in Figure 12b, there are two areas marked with green boxes on sloping road area are detected mistakenly as obstacles when only the estimated height is used in the Algorithm 3. So from the Figure 11 and Figure 12, we can see that the estimated height of road can handle most of the road conditions except sloping road. However, the estimated vector can deal with sloping condition effectively. Therefore, both jointly applied to obstacle extraction to improve the accuracy of the detection.

**Figure 13:**
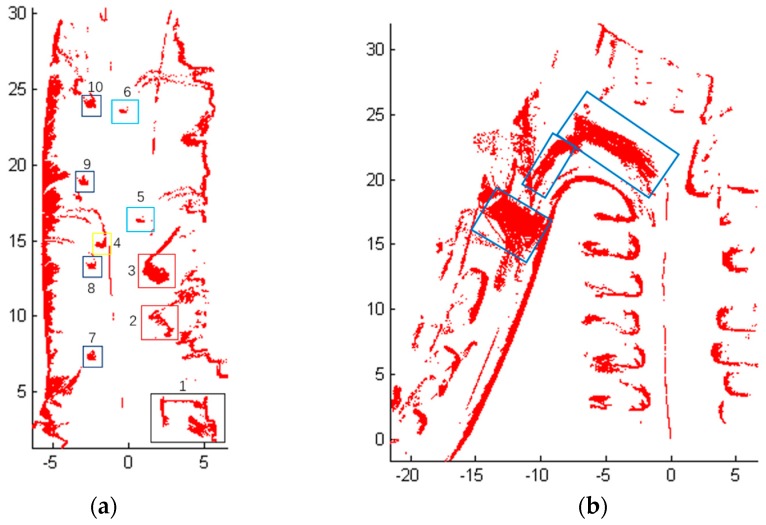
Obstacle extraction by Blas’ method. (**a**) The results of Scene 1; (**b**) The results of Scene 3.

To compare our method with other existing methods, the obstacles in scene 1 and scene 3 were also extracted using Blas’ method [18]. In Blas’ method, the obstacles were extracted using a combined classifier fused four salient features, i.e., terrain height, terrain slope, increments in terrain height, and variance in height across the terrain. The obstacles extracted by Blas’ method are illustrated in Figure 13. Figure 13a is the result of scene 1, although all the obstacles were detected, many noise spots were not removed, because of his method is based on raw point. There are three areas marked with blue boxes on sloping and downhill road are detected mistakenly as obstacles as shown in Figure 13b. It can be seen that Blas’ method cannot be applied to complex environments such as Scene 3. In contrast, our proposed method can adapt to different and changing road conditions, as the estimated road height and road vector can vary with the changing road conditions, and improves its adaptivity.

**Figure 14:**
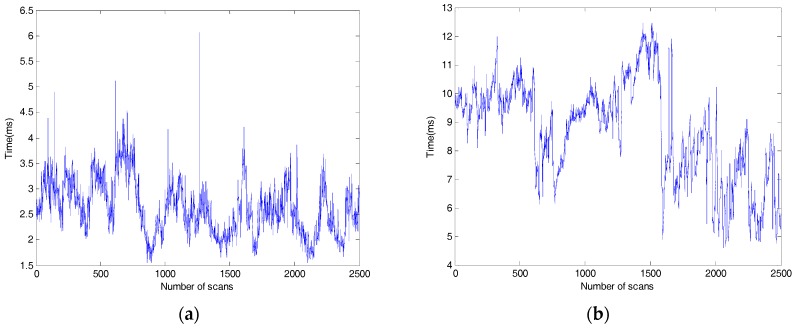
The computing time of our method and Blas’s method. (**a**) Our method; (**b**) Blas’ method.

Figure 14 shows a comparison of the computing time used in Scene 3 between our method and Blas’ method. The average and the worst computational time of our method are about 6.06 ms and 2.62 ms, respectively. The average execution time of Blas’ method is about 8.67 ms, which is higher than the worst computational time of our method. The proposed method can be used to real-time process of mobile LiDAR point clouds for obstacles detecting.

## 6. Conclusions

This paper presents a new approach on the detection of road and obstacles using a single downward-looking 2D LiDAR, which is a very effective and economic solution for unmanned ground vehicles in urban environments. Although it has some limitations in distinguishing dynamic obstacles such as pedestrians, the proposed method shows a stable performance in detecting passable road and static obstacles of different height. The main contribution of our research is that the height and the vector of scanned road surface are estimated iteratively for each frame, and both jointly applied to classify the extracted line segments into road and obstacle line segments. Therefore, our method can detect passable roads and static obstacles at different height and effectively handle the changes of road conditions, e.g. uphill road, downhill road or sloping road. Only the 2D planar position and the steering angle of the robot are used during the whole design and application. The proposed method was tested in both normal scenes and complex scenes. The experimental results show that the proposed algorithms outperform existing methods. All the parameters of our algorithms were empirically turned by simple experiments. 

The proposed method has some limitations in distinguishing dynamic obstacles such as pedestrians. In the future woke, we would research about the determination of adaptive threshold based on the calibration parameters of LiDAR, robot and the input of demand. Moreover, to overcome the limitations in distinguishing dynamic obstacles, we plan to conduct further research on how to reduce the impacts of the appeared dynamic obstacles to enhance the detection accuracy by using both 2D LiDAR and camera-based target recognition.

## Figures and Tables

**Table 1:** An overview of related works.

Reference	Sensor	Method	Advantages	Disadvantages
Zermas et al. [12]	3D LiDAR	Iterative fashion using seed points	Rich information of obstacles	Expensive and height processing time
Himmelsbach et al. [13]	3D LiDAR	Establishing binary labeling		
Chen et al. [14]	Horizontally-looking 2D LiDAR	Based on occupancy grid map	Simple principle	The obstacles that are lower than the scanning height can not be detected
Chung et al. [15]	Horizontally-looking 2D LiDAR	Support vector data description	No geometric assumption and the robust tracking of dynamic object	
Lee et al. [19]	Downward-looking 2D LiDAR	Quantized digital elevation map and grayscale reconstruction	Data processing by using existing image processing techniques	Not discuss the conversion between different scene
Andersen et al. [22]	Downward-looking 2D LiDAR	Terrain classification based on derived models	Convenient and direct	Poorly suited to the changing conditions
Liu et al. [26]	Downward-looking 2D LiDAR	Dynamic digital elevation map	Adaptive curb model selection	Not discuss complex road conditions

**Table 2:** Configuration Parameters for LiDAR Sensor.

Parameter	Description	Value
α	titled down angle	8°
$\theta_{\min}$	start scanning angle	15°
$\theta_{\max}$	stop scanning angle	165°
Δθ	angular resolution	0.5°

**Table 3:** Parameters for Obstacle Detection.

Parameter	Description	Value
λ	auxiliary parameter	10°
$\sigma_{l}$	residual variance	0.02 m
κ	the minimum number of point	8
$\delta_{th}$	height threshold of point	0.15 m
$\phi_{\max}$	direction angle threshold	15°
$L_{\min}$	length threshold of line	0.4 m
$l_{\min}$	segment threshold of noise	0.0001 m
$\xi_{h}$	height threshold of line	0.14 m
ς	deviation	0.2 m
